# CT‐based estimation of medial and lateral femoral joint lines in extension and flexion: A large three‐dimensional validation study

**DOI:** 10.1002/jeo2.70764

**Published:** 2026-06-22

**Authors:** Hannes Vermue, Liudi Yang, Gianna Scire, Jason Otto, Cécile Batailler, Sébastien Lustig

**Affiliations:** ^1^ Department Orthopedic Surgery University Hospital Ghent Ghent Belgium; ^2^ Department of Orthopedic Surgery and Sport Medicine, Croix‐Rousse Hospital FIFA Medical Center of Excellence Lyon France; ^3^ Stryker Mahwah New Jersey USA; ^4^ Université de Lyon Université Claude Bernard Lyon 1 Lyon France

**Keywords:** image‐based, joint line, revision, robotic‐assisted, total knee arthroplasty

## Abstract

**Purpose:**

Successful total knee arthroplasty relies on precise joint line restoration, but anatomical variations and bone loss complicate this intraoperatively. This study aimed to develop computed tomography (CT)‐based estimates of the medial and lateral femoral joint lines in extension and flexion using three‐dimensional imaging.

**Methods:**

Using a Computed Tomography database, the medial and lateral joint lines in extension and in flexion relative to the medial and lateral epicondyles were analysed in 724 nondegenerative femora. Linear regression models were developed using femoral width as the predictor. Independent validation was performed in 264 femora, not included in the regression analyses, to analyze prediction accuracy.

**Results:**

The linear regression models predicted the medial and lateral extension joint lines with an *R*² of 70.1% and 73.0%, respectively. Similarly, *R*² values of 48.8% and 52.6% were obtained for the medial and lateral flexion joint lines. Validation of the models resulted in joint line prediction both in extension and in flexion, with an average error from 0 to 0.3 mm, and the 95% confidence intervals ranged up to approximately ±3 mm.

**Conclusion:**

This study presents CT‐based regression models that characterise native femoral joint line anatomy using a large cohort of nondegenerative femora. By defining reproducible relationships between femoral geometry and joint line position, the proposed approach provides imaging‐derived reference values that may support preoperative planning and joint line estimation. These findings contribute to a more detailed anatomical understanding of joint line morphology and offer a quantitative framework that could be integrated into image‐based planning workflows, although clinical validation is still required.

**Level of Evidence:**

Level IV.

## Graphical abstract

One sentence summary: A computed tomography‐based femoral width regression model estimated distal and posterior joint line references with all validation errors remaining within the clinically relevant 4‐mm threshold.

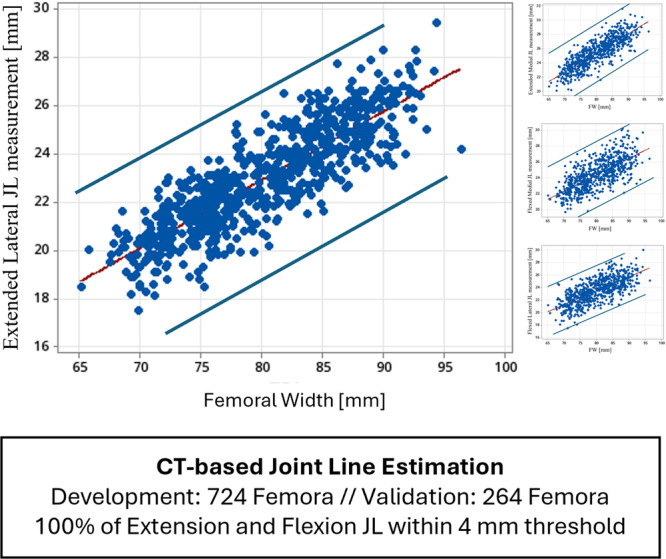

**Abbreviations**
3D: three‐dimensional
AP: anteroposterior
CI: confidence interval
CT: computed tomography
ELJL: extended lateral joint line
EMJL: extended medial joint line
FLJL: flexed lateral joint line
FMJL: flexed medial joint line
FW: femoral width
JL: joint line
LE: lateral epicondyle
MA: mechanical axis
ME: medial epicondyle
ML: mediolateral
MRI: magnetic resonance imaging
PCOR: posterior condylar offset ratio
SOMA: Stryker orthopaedics modelling and analytics
TEA: transepicondylar axis
TKA: total knee arthroplasty

## INTRODUCTION

Total knee arthroplasty (TKA) reliably relieves pain and restores function in end‐stage osteoarthritis. However, a clinically meaningful proportion of patients remain dissatisfied postoperatively [6]. Among multiple contributors, inaccurate restoration of the native joint line has been consistently associated with altered knee biomechanics, instability and worse patient‐reported outcomes [4, 15, 20]. Clinical studies suggest that deviations exceeding 4 mm from the pre‐arthritic joint line increase the risk of suboptimal results [15, 17]. Consequently, patient‐specific methods are needed to estimate the joint line in both extension (distal articular level) and flexion (posterior articular level).

Conventional practice typically relies on fixed distances from bony landmarks (e.g., epicondyles, adductor tubercle, fibular head) or on simple ratios that scale with femoral size [12, 19, 27, 30]. However, these approaches are vulnerable to landmark identification errors [26, 31, 36], and to distortion of anatomy due to bone loss, or deformity, particularly in complex primary and revision TKA. In addition, estimation of the joint line in flexion presents specific challenges, as posterior condylar references may be affected by wear in advanced osteoarthritis or by imperfections of a prior implant in revision settings [5, 22]. These shortcomings motivate a data‐driven, three‐dimensional (3D) approach.

High‐resolution CT‐based 3D modelling enables precise, reproducible landmark detection and quantitative assessment across large, heterogeneous populations [28, 29, 33]. Prior work has largely focused on the extension joint line or on 2D measurements, and typically reports aggregate (non‐compartment‐specific) estimates [1, 8, 10, 12, 16, 18, 19, 25, 27, 30, 32]. There is a need for validated algorithms that (i) provide separate medial and lateral joint line estimations, (ii) cover both extension and flexion and (iii) are derived from large, multicenter datasets to improve generalisability. Defining such relationships requires nonosteoarthritic anatomy, as osteoarthritic remodelling alters the native joint line and limits the ability to establish true anatomical reference values.

Therefore, the study aimed to develop and validate regression algorithms that estimate the medial and lateral joint lines in extension and in flexion from distal nonosteoarthritic femoral geometry on 3D CT. It was hypothesised that a large, high‐quality CT dataset would (1) yield stronger predictive performance than previously reported and (2) provide clinically interpretable error bounds relative to the 4‐mm threshold associated with dissatisfaction. While the 4‐mm threshold originates from studies evaluating the joint line in extension, its application in flexion should be considered a contextual benchmark rather than a validated clinical cutoff.

## METHODS

The current study utilised the Stryker orthopaedics modelling and analytics (SOMA) database, a CT‐scan‐based modelling and analytics system composed of scans of over 25,000 bones. The SOMA system, including scans without motion artifacts and a slice thickness of 1.5 mm or less, has been shown to provide accurate measurements with less deviation than human‐mapped points [29]. In addition, these measurements are highly repeatable with a demonstrated measurement variation of 0.2%. Of the scans, 724 left femora were used for model development to maximise the precision and stability of the regression estimates. These femora met predefined eligibility criteria, including high‐resolution CT imaging and absence of CT‐detectable osseous degenerative changes (osteophytes, subchondral sclerosis, cystic changes or deformity of the distal femoral articular surface). For independent validation, 264 right femora meeting the same criteria were identified from a nonoverlapping set of patients. The use of contralateral‐sided femora for validation reflected dataset availability after filtering and ensured a strict separation between the development and validation cohorts. The dataset included models from individuals of Caucasian, African, Asian, Middle Eastern and Unknown ethnicity. There were 339 females and 385 males in the study ranging from age 18 to 94. The SOMA database is an industry‐maintained repository of fully anonymised CT‐based bone models derived from patients who provided prior informed consent for the use of their imaging data in research. No identifiable patient information was available to the investigators, and no additional imaging, data collection or patient contact was performed as part of this study. As the analysis was limited to secondary use of anonymised data, separate ethical committee approval was not obtained.

### Coordinate system

The coordinate system used in this study was constructed by (1) the femoral mechanical axis (MA): the axis connecting the femoral head centre and the Facies Patellaris saddle point, (2) the anteroposterior (AP) axis: the cross product of the femoral MA and the transepicondylar axis (TEA) and (3) the mediolateral (ML) axis: the cross product of the AP axis and the femoral MA.

### Determination of femoral width (FW)

The medial epicondyle (ME), defined as the medial sulcus on the ME, and the lateral epicondyle (LE), defined as the most lateral point on the LE, are preset landmarks in the SOMA system. They were points picked on a template bone shape, which were then mapped onto the patient bone with a transformation [26]. The template bone point selections for the epicondyles were confirmed by surgeons. Superior precision was demonstrated with the automated method compared to manual identification of the femoral epicondyles, with errors up to 2 mm instead of up to 4 mm [29]. The point‐to‐point distance between the two epicondyles defined the FW.

### Calculating joint line

**Figure 1:**
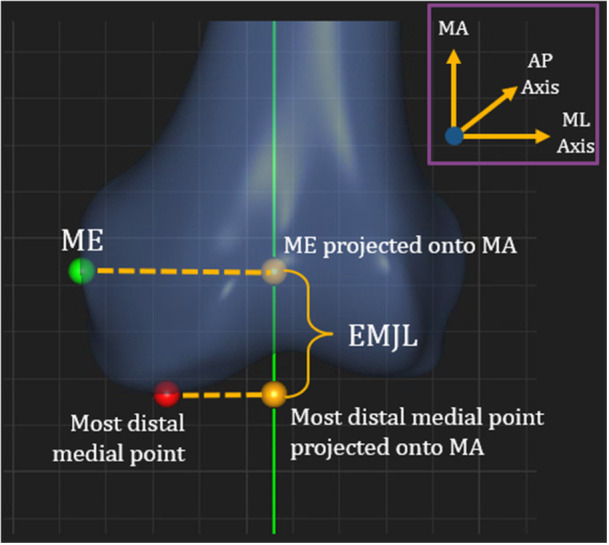
EMJL calculation in SOMA. AP, anteroposterior; EMJL, extended medial joint line; MA, mechanical axis; ME, medial epicondyle; ML, Mediolateral; SOMA, Stryker orthopaedics modelling and analytics.

For each femur in the study cohort, the following joint line calculations were made: (1) the extended medial joint line (EMJL), (2) the extended lateral joint line (ELJL), (3) the flexed medial joint line (FMJL) and (4) the flexed lateral joint line (FLJL). With the femur oriented in the coronal view (the ML axis as the *x*‐axis and the MA as the *y*‐axis), the distance between the ME/LE and the medial/lateral extended (distal) joint line location parallel to the MA was determined. Figure 1 shows an example of how the EMJL is calculated. This method was repeated on the lateral side as well to obtain the ELJL.

**Figure 2:**
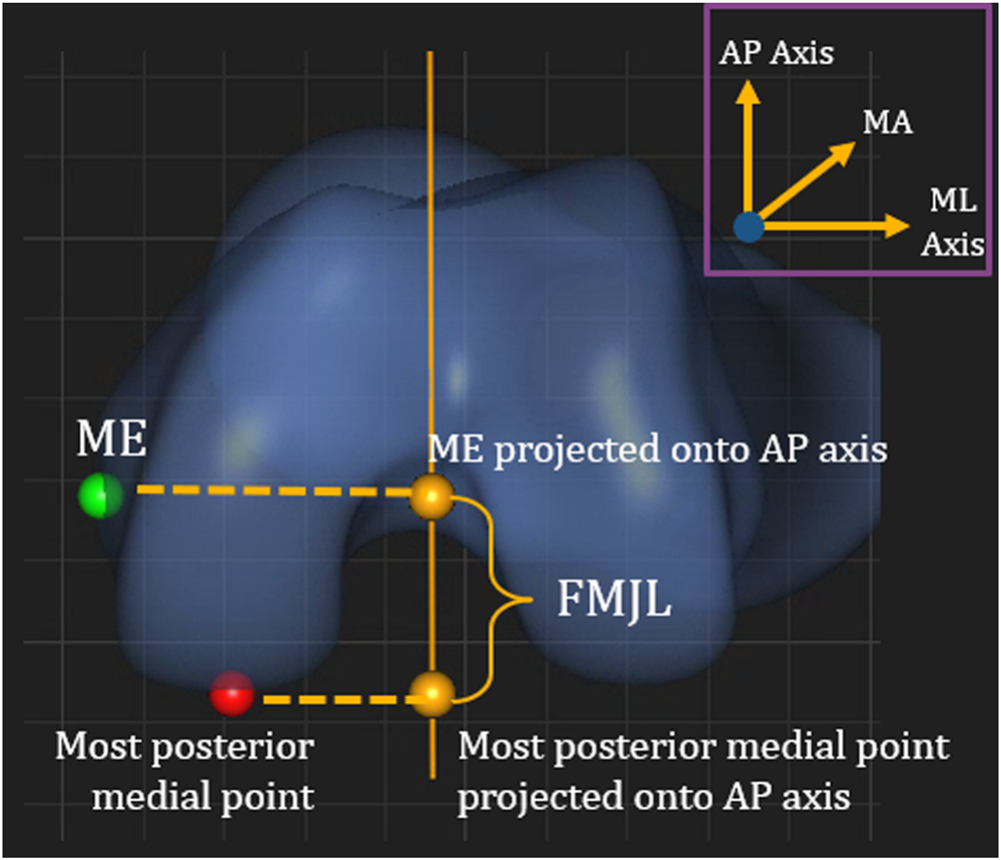
FMJL calculation in SOMA. AP, anteroposterior; FMJL, flexed medial joint line; MA, mechanical axis; ME, medial epicondyle; ML, mediolateral; SOMA, Stryker orthopaedics modelling and analytics.

For the flexed joint lines, the femur was oriented in the transverse view (the ML axis as the *x*‐axis and the AP axis as the *y*‐axis) and the distance between the ME/LE and the medial/lateral flexed (posterior) joint line location parallel to the AP axis was determined. Figure 2 shows an example of how the FMJL is calculated. This method was completed on the lateral side as well to obtain the FLJL.

### Linear regression for joint line prediction

A linear relationship was found between FW and each of the four joint line measurements. A linear regression equation was calculated for each of the four joint lines with FW as the predictor (x) and the joint lines as the response (y).

### Tolerance interval for joint line prediction

**Figure 3:**
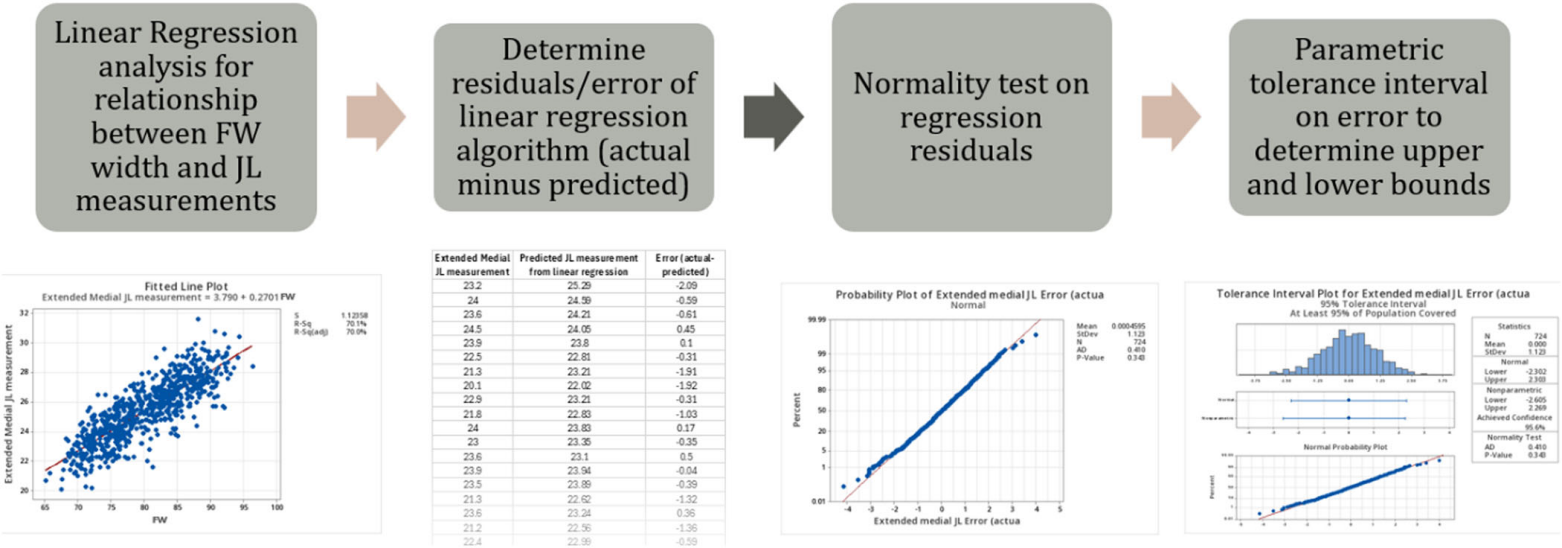
Summary of statistical analysis. FW, femoral width; JL, joint line.

For each regression equation, residuals were calculated as the difference between the actual and predicted joint line measurements and were then evaluated for normality. The Anderson‐Darling or Ryan‐Joiner normality test showed all four sets of residuals came from a normally distributed population. Therefore, the tolerance interval for a normal distribution was calculated for each set of residuals to get the upper and lower bounds of each linear regression model with 95% confidence for 95% of the population. A summary of the statistical analysis steps is shown in Figure 3 in a flowchart format. Minitab® Version 21.1 (Minitab LLC) was used for the statistical analysis.

### Algorithm validation

Validation of the linear regression equations was performed using 264 right femora from a cohort with no patient overlap with the initial 724 cases. The average and 95% confidence intervals (CI) were obtained for the difference between the actual joint lines and the predicted joint lines.

### Comparison to conventional ancillary to assess extension joint line

One of the currently available guides to assess the femoral joint line in extension consists of an intramedullary canal‐based guide, which estimates the joint line at 28 mm from the ME. It takes into account a fixed 6° valgus angulation between the intramedullary canal and the femoral MA. This instrument was based on a cadaveric assessment, which found 28 mm to be the mean distance between the ME and JL [23]. The cutting guide was recreated in SOMA, and the predicted extended medial and lateral joint lines were calculated and then compared with the actual patient joint line measurements to obtain the average error. This comparison allowed comparison between a widely used instrument and the proposed SOMA regression predictions.

## RESULTS

**Figure 4:**
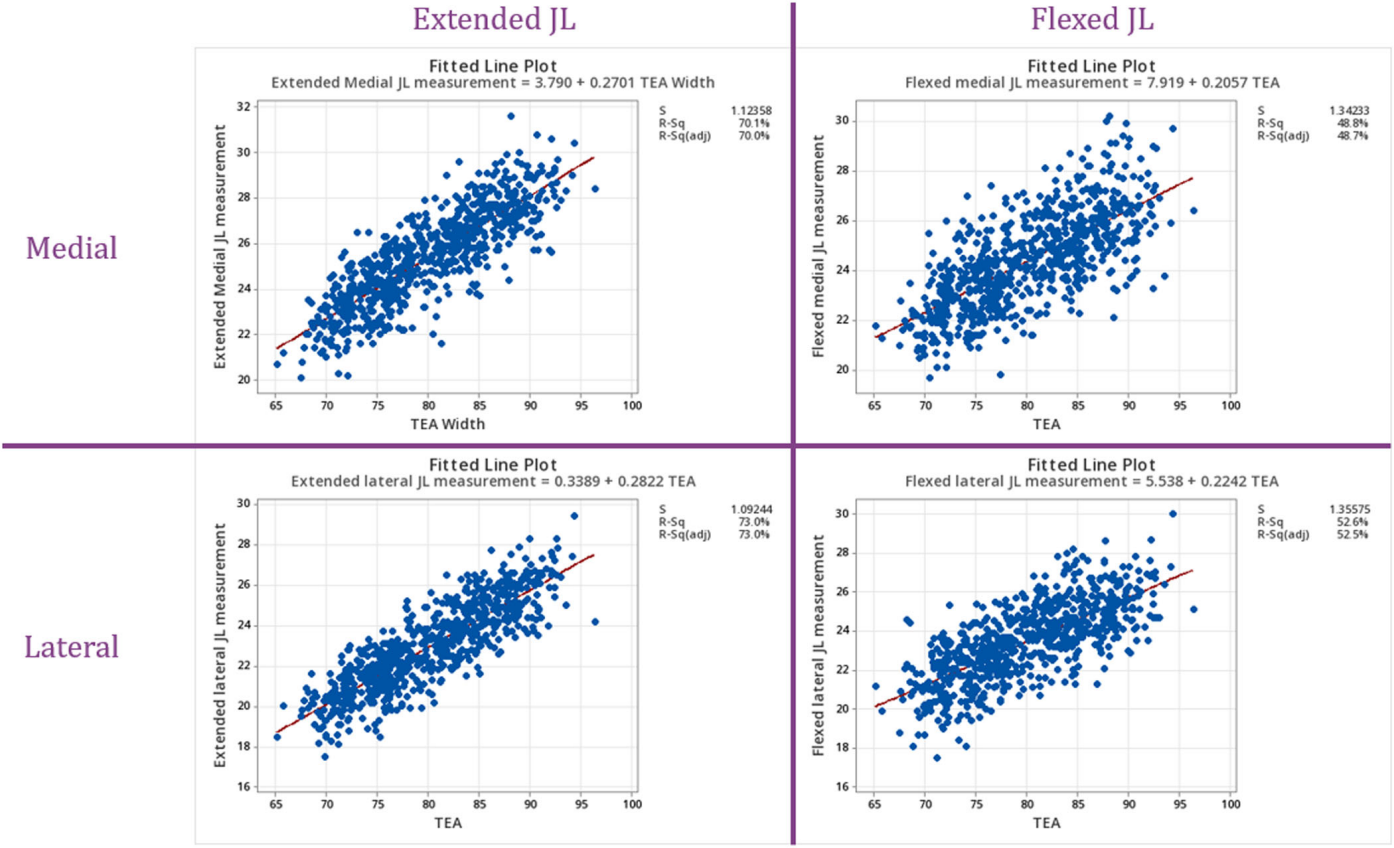
Linear regression for four JL measurements. JL, joint line; TEA, Transepicondylar axis.

**Figure 5:**
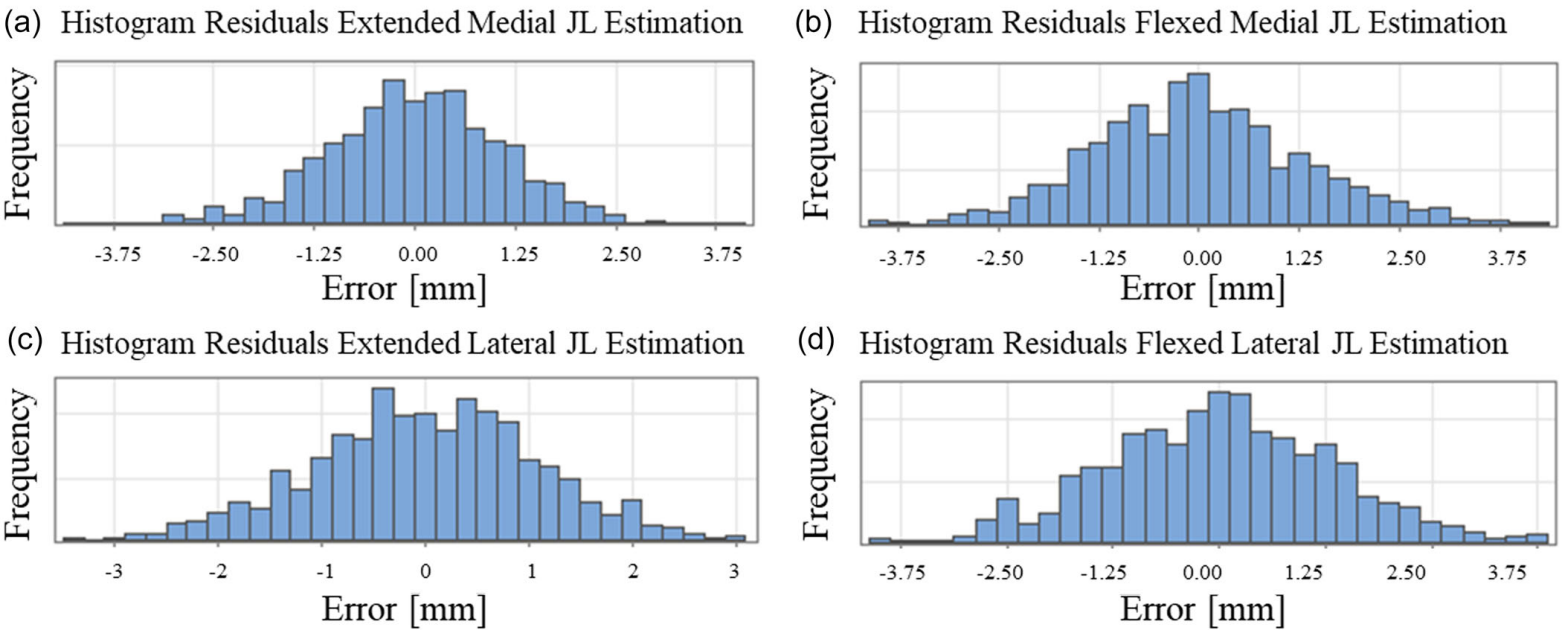
The frequency of the residuals of each linear regression equation is visualised. (a) extended medial joint line; (b) flexed medial joint line; (c): extended lateral joint line; (d) flexed lateral joint line. JL, joint line.

Of the 724 femora included in the linear regression analysis, 385 were obtained from men and 339 from women. Based on these samples, the linear regression equations shown in Table 1 were derived (Figure 4). The coefficient of determination (*R*²) ranged from 70.1% to 73.0% for the joint line in extension and from 48.8% to 52.6% for the joint line in flexion, reflecting moderate to strong model fit. The residuals of each regression followed a normal distribution (Figure 5). To validate the algorithm, the remaining 264 femora not used for model generation were analysed by calculating the error (actual—predicted) for each of the four joint line measurements. The mean error and corresponding 95% CIs are summarised in Table 2, with average errors ranging from –0.3 mm (95% CI –3.2 to 2.8) for the FLJL to 0.3 mm (95% CI –2.4 to 3.0) for the ELJL. In the validation cohort, deviations of the algorithmic joint line reconstruction exceeded the clinically relevant threshold of 4 mm in none of the cases, either medially or laterally.

**Table 1:** The results of the linear regression analysis are displayed for each analysed joint line.

Joint Line	Equation	95% CI	*R*²
Extended medial	3.79 + 0.2701 * [FW]	[−2.4; 2.4]	70.1%
Extended lateral	0.3389 + 0.2822 * [FW]	[−2.3; 2.3]	73.0%
Flexed medial	7.919 + 0.2057 * [FW]	[−2.8; 2.8]	48.8%
Flexed lateral	5.538 + 0.2242 * [FW]	[−2.8; 2.8]	52.6%

*Note*: The unit of the Femoral Width is millimetre. Abbreviations: CI, confidence interval; FW, femoral width.

**Table 2:** The results of the validation dataset are visualised for each analysed joint line.

Measurement	Average error [mm]	95% CI	> 4 mm of native JL
Extended medial JL	0.0	[−2.5; 2.5]	0% (0/264)
Extended lateral JL	−0.2	[−2.6; 2.3]	0% (0/264)
Flexed medial JL	0.3	[−2.4; 3.0]	NA
Flexed lateral JL	−0.3	[−3.2; 2.8]	NA

Abbreviations: CI, confidence interval; JL, joint line; mm, millimetre.

### Comparison to conventional ancillary to assess extended joint line

The average errors of the predicted extended medial and lateral joint by the intramedullary canal referencing distal resection guide were −2.8 mm (95% CI [−6.8; 1.0]) and −5.6 mm (95% CI [−10.0; −0.6]) respectively (Table 3). This shows that the manual instrumentation generally distalizes the joint line for most patients. In the validation group, the error on joint line reconstruction with the conventionally ancillary exceeded the clinically relevant threshold of 4 mm in 33% (86 out of 264) on the extended medial JL and 75% (198 out of 264) on the extended lateral JL.

**Table 3:** Overview of the average error and 95% CI of the intramedullary canal‐referencing distal resection guide.

Measurement	Average error [mm]	95% CI	> 4 mm of native JL
Extended medial JL	−2.8	[−6.8; 1.0]	33% (86/264)
Extended lateral JL	−5.6	[−10.0; −0.6]	75% (198/264)

Abbreviations: CI, confidence interval; JL, joint line; mm, millimetre.

## DISCUSSION

This study represents the largest cohort to date evaluating the native knee joint line in extension and flexion and provides imaging‐based reference relationships for joint line estimation. The models also include flexion joint line estimates, which have been less frequently reported in prior work. The present work should be interpreted as an imaging‐based anatomical reference study. No intraoperative validation, implant‐position analysis or postoperative functional assessment was performed. Therefore, no conclusions can be drawn regarding improved joint line restoration, kinematics or clinical outcomes, and these aspects require dedicated prospective evaluation. The proposed model may provide anatomical reference values that could potentially be incorporated into image‐based planning workflows, but its clinical utility remains to be demonstrated.

Restoration of the prearthritic joint line remains important in TKA, as deviations exceeding 4 mm have been associated with worse outcomes [17]. The extension joint line has been thoroughly evaluated; the ratio of the Adductor Tubercle to EMJL (ATEMJL)/FW has been reported as a reliable reference [3, 9, 12, 13, 19, 21, 35]. In this study, the medial and lateral joint lines were defined based on the medial and lateral femoral epicondyles, respectively. Although no direct comparison was made between the ATEMJL/FW and MEEMJL/FW ratios, other research has shown ATEMJL correlates better with FW [8, 9, 12, 13, 19, 21]. The extension joint line models demonstrated *R*² values within the upper range of those previously reported using ATEMJL/FW‐based approaches [3, 9, 19, 21, 35]. Moreover, these *R*²‐values are higher than those in previous studies reporting MEEMJL or LEEMJL/FW ratios on AP radiographs or intraoperative measurements. Using CT rather than MRI or plain radiography, the present analysis yielded regression coefficients of 0.27 and 0.28 for MEEMJL/FW and LEEMJL/FW, respectively. These align with other studies reporting similar ranges of 0.24–0.395 and 0.28–0.32 for ME and LEs, although no clear superiority can be demonstrated over other established methods for joint‐line estimation [1, 10, 11, 13, 18, 19, 25, 27, 30, 32]. Importantly, multiple studies show sex does not influence the ratios used to estimate the joint line level [8, 13, 19, 25].

Unlike most prior studies, the present methodology defines medial and lateral joint lines separately. This distinction may be relevant in revision settings and in complex primary TKA. Several studies mimic revision settings using a fixed valgus angle relative to the femur′s intramedullary canal to approximate the MA, while others measure perpendicular to the joint line, risking variations due to joint line obliquity [19]. Conventional instruments with joint line approximation tools are also used in revision settings with a fixed 28 mm reference from the ME, again using a fixed valgus angle. While mechanical alignment is usually the target in revisions compared to personalised approaches in primary TKAs, the ability to predict medial and lateral joint lines separately matters in both [14]. The inferior performance of conventional ancillary joint line estimation in this study, with a significant proportion of cases exceeding 4 mm, may reflect differences between standardised SOMA landmarking and the nonstandardised ME definitions used in earlier cadaveric studies [23].

Another asset of this study is flexion joint line estimation, a feature absent in most prior studies [8, 10, 16, 25, 30]. Griffin et al. and Servien et al. used a single MRI slice to define FW and epicondyle distances to the posterior joint line [10, 30]. The study by Griffin et al reported only the absolute distances of the epicondyles to the posterior joint line, whereas the study by Servien et al provided ratios of 0.29 (SD 0.03) and 0.28 (SD 0.03) for MEFMJL or LEFLJL over the FW, respectively, which were numerically higher compared to the present study [10, 30]. Unfortunately, no correlation coefficients were reported in their analyses. Both femoral epicondyles might not be fully visible on a single axial MRI slice, which could introduce a systematic error. The study by Lee et al. used 3D models of the distal femur, which demonstrated comparable linear regression values, but lower *R*² values compared to the present study for prediction of the posterior joint lines [16]. Similarly, Fan and colleagues defined the joint lines in a Chinese population, reporting lower *R*² values for the extension joint lines, but higher *R*²‐values for the medial posterior joint line [8]. In a Turkish study on 40 cadavers, Ozkurt et al reported the results of their linear regression analysis with lower *R*² values for extension joint line estimation than the present study, though they found slightly higher *R*² values for flexion joint line estimation [25].

Although flexion joint line estimation equations have been published, clinical practice often relies on restoring the PCOR from the native femur or a prior implant. However, the original anatomy may be altered due to bony deformity in cases of severe osteoarthritis posing difficulties to achieve the prearthritic joint line level. In the revision setting, restoring the native PCOR relies on the previous implant maintaining the prearthritic joint line. The proposed algorithm may provide an adjusted joint line reference when PCOR is unreliable, but clinical validation will be needed [2]. The lower *R*² values observed for flexion joint line estimation likely reflect the greater anatomical variability of posterior femoral morphology and its weaker linear relationship with FW. In addition to this morphological variability, knee kinematics may further limit the predictability of the flexion joint line, as the relative position of the posterior femoral condyles during flexion is influenced by joint motion characteristics rather than being determined solely by static femoral geometry [7, 24, 34]. The interaction between posterior morphology and knee kinematics, therefore, likely contributes to the lower coefficients of determination observed for flexion compared with extension joint line estimation.

One of the limitations of previous research on joint line preservation is the limited intraoperative application of imaging‐based ratios. Although regression analyses provide reproducible estimates for defining the joint line, its implementation in real‐time during surgery has been minimal, requiring the surgeon to manually measure distances and estimate anatomical landmarks. This manual process introduces variability, as precise identification of anatomical landmarks and consistent measurement accuracy can be challenging in an operative setting [26, 31, 36]. The complexity required for such calculations particularly in the high‐pressure environment of surgery cannot be underestimated.

As intraoperative implementation was not evaluated in this study, no conclusions can be drawn regarding the impact of this approach on surgical planning or joint line restoration. Nevertheless, compared to prior ratio‐based methods with limited intraoperative applicability, the proposed regression‐based model may be more amenable to integration within image‐based surgical workflows. As image‐based navigation and robotic systems can provide joint line estimates before bone resection, they potentially could improve surgical planning and reduce the risk of joint line malposition. Ongoing advances in automated segmentation and landmark identification are expected to further reduce the manual burden associated with joint line estimation [28, 29].

One of the potential limitations of the present analysis is the use of a proprietary database. However, the resulting regression equations are based on generic anatomical measurements rather than SOMA‐specific features. As such, the approach is conceptually transferable to other imaging pipelines capable of reliably extracting FW and epicondylar landmarks. Nevertheless, independent validation using nonproprietary datasets will be important to further establish reproducibility and generalisability. Similarly, the model was validated on femora without CT‐detectable osseous features of degenerative arthritis, rather than on knees with established osteoarthritis. It does not guarantee similar performance across different populations or pathologic conditions, potentially limiting external validity. While this means the algorithm reflects predisease anatomy, this characteristic can be advantageous as its precision on healthy femora provides a reliable anatomical baseline that helps surgeons approximate where the patient′s joint line presided prior to deformity. For knees with advanced osteoarthritic changes, this baseline can serve as a valuable reference, offering a consistent and accurate method for restoring the estimated joint line. Another limitation to consider is that this study relies on imaging data that may not always be available, depending on the surgical setting. Preoperative or intraoperative imaging requires specific equipment, which may limit the algorithm′s broad application, particularly in settings where such resources are unavailable. However, wider availability of intraoperative imaging technology may increase the feasibility of implementing such approaches across surgical centres in the future. The large, multicenter dataset may introduce unmeasured heterogeneity (scanner protocols, demographics), which should be acknowledged as a limitation as well.

## CONCLUSION

This study presents CT‐based regression models for femoral joint line estimation derived from structurally preserved femoral anatomy. The proposed approach may support imaging‐based prediction and preoperative planning by providing patient‐specific joint line reference values, with potential to help avoid excessive bone resection and to inform personalised alignment strategies. However, the findings are limited to nondegenerative femora and imaging‐based estimation, and further studies are required to assess applicability in osteoarthritic knees and clinical planning workflows.

## AUTHOR CONTRIBUTIONS

**Vermue Hannes**: Data collection and analysis; writing manuscript. **Liudi Yang**: Data collection and analysis; writing and review of manuscript. **Gianna Scire**: Data collection and analysis; writing and review of manuscript. **Jason Otto**: Data collection and analysis; writing and review of manuscript. **Cécile Batailler**: Supervision; Interpretation and writing manuscript. **Sébastien Lustig**: Supervision; interpretation and writing manuscript.

## FUNDING INFORMATION

The authors have no funding to report.

## CONFLICT OF INTEREST STATEMENT

Vermue Hannes and Cécile Batailler declare that they have no conflicts of interest. Liudi Yang, Gianna Scire and Jason Otto: Paid employee for Stryker. Sébastien Lustig: Consultant for Stryker, Smith and Nephew, Heraeus. Institutional research support from Lepine and Amplitude. Editorial Board for Journal of Bone and Joint Surgery (Am).

## ETHICS STATEMENT

No ethical committee approval was necessary, as a database owned by Stryker (Stryker Orthopaedics Modelling and Analytics [SOMA]) was used with anonymised patient data who have given prior consent to use their data for further scientific work.

## Data Availability

The data that support the findings of this study are available from the corresponding author upon reasonable request.

## References

[1] Aljuhani WS , Alsaeed AA , Alrashed MO , Alanazi AM , Alsalman MJ . Lateral epicondyle to the joint line distance is a precise landmark for determination of an accurate knee joint line: an observational retrospective study. J Exp Orthop. 2023;10:62. 37289300 10.1186/s40634-023-00621-z PMC10250280

[2] Almeida PH , Vilaça A . The posterior condylar offset ratio and femoral anatomy in anterior versus posterior referencing total knee arthroplasty. Orthop Traumatol Surg Res. 2015;101:687–691. 26205566 10.1016/j.otsr.2015.05.003

[3] Boya H , Şükrü ARAÇ S . Does severe osteoarthritis in knees with varus deformity alter the adductor ratio? Acta Orthop Traumatol Turc. 2017;51:437–441. 29029868 10.1016/j.aott.2017.09.007 PMC6197182

[4] Carpenter CW , Cummings JF , Grood ES , Leach D , Paganelli JV , Manley MT . The influence of joint line elevation in total knee arthroplasty. Am J Knee Surg. 1994;4:164–167.

[5] Clement ND , MacDonald DJ , Hamilton DF , Burnett R . Posterior condylar offset is an independent predictor of functional outcome after revision total knee arthroplasty. Bone Jt Res. 2017;6:172–178. 10.1302/2046-3758.63.BJR-2015-0021.R1 PMC5376661 28360083

[6] DeFrance MJ , Scuderi GR . Are 20% of patients actually dissatisfied following total knee arthroplasty? A systematic review of the literature. J Arthroplast. 2023;38:594–599. 10.1016/j.arth.2022.10.011 36252743

[7] Dobbelaere A , Müller JH , Aït‐Si‐Selmi T , Gousopoulos L , Saffarini M , Bonnin MP . Sagittal femoral condylar shape varies along a continuum from spherical to ovoid: a systematic review and meta‐analysis. Arch Orthop Trauma Surg. 2023;143:3347–3361. 36121475 10.1007/s00402-022-04613-z

[8] Fan A , Xu T , Li X , Li L , Fan L , Yang D , et al. Using anatomical landmarks to calculate the normal joint line position in Chinese people: an observational study. J Orthop Surg Res. 2018;13:261. 30340645 10.1186/s13018-018-0963-2 PMC6194602

[9] Gao Z , Mao X , Xiang C , Gao Y , Zhang X , Guo Z . An accurate method for locating the joint line during revision total knee arthroplasty: a radiologic study in the Chinese population. Knee. 2021;29:510–519. 33756261 10.1016/j.knee.2021.03.003

[10] Griffin FM , Math K , Scuderi GR , Insall JN , Poilvache PL . Anatomy of the epicondyles of the distal femur. J Arthroplast. 2000;15:354–359. 10.1016/s0883-5403(00)90739-3 10794232

[11] Hou Y , Jiang J , Liu H , Wang R , Wu J , Wang Y , et al. Identification of the joint line in revision total knee arthroplasty using a multiple linear regression model: a cadaveric study. Arch Orthop Trauma Surg. 2023;143:5239–5248. 36971801 10.1007/s00402-023-04792-3

[12] Iacono F , Lo Presti M , Bruni D , Raspugli GF , Bignozzi S , Sharma B , et al. The adductor tubercle: a reliable landmark for analysing the level of the femorotibial joint line. Knee Surg Sports Traumatol Arthrosc. 2013;21:2725–2729. 22744435 10.1007/s00167-012-2113-4

[13] Iacono F , Raspugli GF , Bruni D , Filardo G , Zaffagnini S , Luetzow WF , et al. The adductor tubercle as an important landmark to determine the joint line level in total knee arthroplasty: from radiographs to surgical theatre. Knee Surg Sports Traumatol Arthrosc. 2014;22:3034–3038. 24362919 10.1007/s00167-013-2809-0

[14] Kafelov M , Batailler C , Shatrov J , Al‐Jufaili J , Farhat J , Servien E , et al. Functional positioning principles for image‐based robotic‐assisted TKA achieved a higher Forgotten Joint Score at 1 year compared to conventional TKA with restricted kinematic alignment. Knee Surg Sports Traumatol Arthrosc. 2023;31:5591–5602. 37851026 10.1007/s00167-023-07609-3

[15] Koshire S , Mohanty SS , Keny SA , Rai AK , Rathod TN , Kamble P . The influence of joint line restoration on functional outcome after primary total knee arthroplasty: a prospective study. J Clin Orthop Trauma. 2022;34:102023. 36161062 10.1016/j.jcot.2022.102023 PMC9490095

[16] Lee M , Ho JPY , Chen JY , Ng CK , Yeo SJ , Merican AM . The relationship of transepicondylar width with the distal and posterior femoral condyles and its clinical implications: a three‐dimensional study. J Knee Surg. 2022;35(3):280–287. 32629512 10.1055/s-0040-1713733

[17] van Lieshout WAM , Valkering KP , Koenraadt KLM , van Etten‐Jamaludin FS , Kerkhoffs GMMJ , van Geenen RCI . The negative effect of joint line elevation after total knee arthroplasty on outcome. Knee Surg Sports Traumatol Arthrosc. 2019;27:1477–1486. 30109369 10.1007/s00167-018-5099-8 PMC6527530

[18] Lutz B , Polcikova L , Faschingbauer M , Reichel H , Bieger R . The epicondylar ratio can be reliably determined in both computed tomography and X‐ray. Arch Orthop Trauma Surg. 2022;142:1185–1188. 33839911 10.1007/s00402-021-03888-y PMC9110527

[19] Luyckx T , Beckers L , Colyn W , Vandenneucker H , Bellemans J . The adductor ratio: a new tool for joint line reconstruction in revision TKA. Knee Surg Sports Traumatol Arthrosc. 2014;22:3028–3033. 25135279 10.1007/s00167-014-3211-2

[20] Luyckx T , Vandenneucker H , Ing LS , Vereecke E , Ing AV , Victor J . Raising the joint line in TKA is associated with mid‐flexion laxity: a study in Cadaver knees. Clin Orthop Relat Res. 2018;476:601–611. 29443845 10.1007/s11999.0000000000000067 PMC6260050

[21] Maderbacher G , Keshmiri A , Schaumburger J , Springorum H‐R , Zeman F , Grifka J , et al. Accuracy of bony landmarks for restoring the natural joint line in revision knee surgery: an MRI study. Int Orthop. 2014;38:1173–1181. 24570152 10.1007/s00264-014-2292-3 PMC4037526

[22] Malviya A , Lingard EA , Weir DJ , Deehan DJ . Predicting range of movement after knee replacement: the importance of posterior condylar offset and tibial slope. Knee Surg Sports Traumatol Arthrosc. 2009;17:491–498. 19139846 10.1007/s00167-008-0712-x

[23] Mason M , Belisle A , Bonutti P , Kolisek FR , Malkani A , Masini M . An accurate and reproducible method for locating the joint line during a revision total knee arthroplasty. J Arthroplast. 2006;21:1147–1153. 10.1016/j.arth.2005.08.028 17162174

[24] Meier M , Zingde S , Steinert A , Kurtz W , Koeck F , Beckmann J . What is the possible impact of high variability of distal femoral geometry on TKA? A CT data analysis of 24,042 knees. Clin Orthop Relat Res. 2019;477:561–570. 30762689 10.1097/CORR.0000000000000611 PMC6382181

[25] Ozkurt B , Sen T , Cankaya D , Kendir S , Basarır K , Tabak Y . The medial and lateral epicondyle as a reliable landmark for intra‐operative joint line determination in revision knee arthroplasty. Bone Jt Res. 2016;5:280–286. 10.1302/2046-3758.57.BJR-2016-0002.R1 PMC4969630 27388715

[26] Robinson M , Eckhoff DG , Reinig KD , Bagur MM , Bach JM . Variability of landmark identification in total knee arthroplasty. Clin Orthop Relat Res. 2006;442:57–62. 16394739 10.1097/01.blo.0000197081.72341.4b

[27] Romero J , Seifert B , Reinhardt O , Ziegler O , Kessler O . A useful radiologic method for preoperative joint‐line determination in revision total knee arthroplasty. Clin Orthop Relat Res. 2010;468:1279–1283. 19890683 10.1007/s11999-009-1114-1 PMC2853645

[28] Schmidt W , LiArno S , Khlopas A , Petersik A , Mont MA . Stryker orthopaedic modeling and analytics (SOMA): a review. Surg Technol Int. 2018;32:315–324. 29791698

[29] Schröder M , Gottschling H , Reimers N , Hauschild M , Burgkart R . Automated morphometric analysis of the femur on large anatomical databases with highly accurate correspondence detection. Open Med J. 2014;1:15–22.

[30] Servien E , Viskontas D , Giuffrè BM , Coolican MRJ , Parker DA . Reliability of bony landmarks for restoration of the joint line in revision knee arthroplasty. Knee Surg Sports Traumatol Arthrosc. 2008;16:263–269. 18046537 10.1007/s00167-007-0449-y

[31] Siston RA , Patel JJ , Goodman SB , Delp SL , Giori NJ , Siston RA . The variability of femoral rotational alignment in total knee arthroplasty. J Bone Jt Surg Am Vol. 2005;87:2276–2280. 10.2106/JBJS.D.02945 16203894

[32] Tantavisut S , Amarase C , Ngarmukos S , Tanavalee C , Tanavalee A . Knee joint line related to bony landmarks of the knee: a radiologic study in a Thai population. Knee Surg Relat Res. 2022;34:5. 35168654 10.1186/s43019-022-00135-5 PMC8845375

[33] Victor J , Van Doninck D , Labey L , Innocenti B , Parizel PM , Bellemans J . How precise can bony landmarks be determined on a CT scan of the knee? Knee. 2009;16:358–365. 19195896 10.1016/j.knee.2009.01.001

[34] Weinberg DS , Streit JJ , Gebhart JJ , Williamson DFK , Goldberg VM . Important differences exist in posterior condylar offsets in an osteological collection of 1,058 femurs. J Arthroplast. 2015;30:1434–1438. 10.1016/j.arth.2015.02.027 25783444

[35] Xiao J , Wang S , Chen W , Yang Y , Liu T , Zuo J . A study to assess the accuracy of adductor tubercle as a reliable landmark used to determine the joint line of the knee in a Chinese population. J Arthroplast. 2017;32:1351–1355. 10.1016/j.arth.2016.10.002 27836580

[36] Yau WP , Leung A , Liu KG , Yan CH , Wong LLS , Chiu KY . Interobserver and intra‐observer errors in obtaining visually selected anatomical landmarks during registration process in non‐image‐based navigation‐assisted total knee arthroplasty. J Arthroplast. 2007;22:1150–1161. 10.1016/j.arth.2006.10.010 18078884

